# Early-Stage Psychotherapy Produces Elevated Frontal White Matter Integrity in Adult Major Depressive Disorder

**DOI:** 10.1371/journal.pone.0063081

**Published:** 2013-04-30

**Authors:** Tao Wang, Xiaolan Huang, Peiyu Huang, Dan Li, Fajin Lv, Yong Zhang, Linke Zhou, Deyu Yang, Peng Xie

**Affiliations:** 1 Institute of Neuroscience, Chongqing Medical University, Chongqing, China; 2 Chongqing Key Laboratory of Neurobiology, Chongqing, China; 3 Department of Neurology, 1^st^ Affiliated Hospital at Chongqing Medical University, Chongqing, China; 4 Department of Radiology, 1^st^ Affiliated Hospital at Chongqing Medical University, Chongqing, China; University of Texas Southwestern Medical Center, United States of America

## Abstract

**Background:**

Psychotherapy has demonstrated comparable efficacy to antidepressant medication in the treatment of major depressive disorder. Metabolic alterations in the MDD state and in response to treatment have been detected by functional imaging methods, but the underlying white matter microstructural changes remain unknown. The goal of this study is to apply diffusion tensor imaging techniques to investigate psychotherapy-specific responses in the white matter.

**Methods:**

Twenty-one of forty-five outpatients diagnosed with major depression underwent diffusion tensor imaging before and after a four-week course of guided imagery psychotherapy. We compared fractional anisotropy in depressed patients (n = 21) with healthy controls (n = 22), and before-after treatment, using whole brain voxel-wise analysis.

**Results:**

Post-treatment, depressed subjects showed a significant reduction in the 17-item Hamilton Depression Rating Scale. As compared to healthy controls, depressed subjects demonstrated significantly increased fractional anisotropy in the right thalamus. Psychopathological changes did not recover post-treatment, but a novel region of increased fractional anisotropy was discovered in the frontal lobe.

**Conclusions:**

At an early stage of psychotherapy, higher fractional anisotropy was detected in the frontal emotional regulation-associated region. This finding reveals that psychotherapy may induce white matter changes in the frontal lobe. This remodeling of frontal connections within mood regulation networks positively contributes to the “top-down” mechanism of psychotherapy.

## Introduction

Major depressive disorder (MDD, major depression), a debilitating psychiatric mood disorder, typically presents as persistent dysthymia, anhedonia, apathy, and occasional suicidal ideation and behavior. The lifetime prevalence of MDD is ranges from 1.5% [1] to 16.2% [2] across various diagnostic criteria and ethnicities. MDD exerts a heavy socioeconomic burden on account of increased disability and suicide rates. Although the mechanism of MDD remains uncertain, successful remedies for the disorder have been applied for over half a century. Both antidepressant medication (ADM) and psychotherapy (PT) have been effective in alleviating symptoms. ADM, including monoamine oxidase inhibitors (MAOIs), serotonin-reuptake inhibitors (SSRIs), and serotonin/noradrenaline-reuptake inhibitors (SNRIs), has been used to treat MDD for nearly half a century, and ADM and PT have been proven to be comparably efficacious in treating depression. Through increasing extracellular neurotransmitter activity, ADM modulates neuron activity in receptor-rich areas, mostly in the limbic system. ADM quells symptoms but fails at preventing future episodes [3]. ADM appears to act as a symptom-reliever rather than as a normalizer of pathophysiologic causality. On the other hand, the basic PT techniques involve talk therapy and/or following therapist instructions, which aids individuals in improving their state of mind and/or behavioral patterns. Various PT approaches have been used to treat depression; the most common are cognitive behavioral treatment (CBT) and interpersonal treatment (IPT). Theoretically, PT targets malfunctioning psychological processes, and symptoms are alleviated through the adjustment of biased attention, information processing, and memory [4].

With the advent of modern imaging methods such as positron emission topography (PET), single photon emission computed tomography (SPECT), and magnetic resonance imaging (MRI), scientists are better able to visually assess mechanisms within the brain. Researches have consistently reported structural and functional abnormalities in the amygdala, hippocampus, frontal lobe, and cingulate cortex of depressed brain [5]. Accumulated evidence has also demonstrated that malfunctioning neural circuits are distributed in the neo-cortex and limbic system [6]. These networks mediate emotional and cognitive processes and are therefore responsible for the various manifestations of MDD. It is widely accepted that dysregulated reciprocal interactions between affective and cognitive networks are correlated with mood disorders and some other psychiatric conditions. Disturbances in these psychological processes may contribute to MDD pathogenesis. An early study first reported increased blood flow in the posterior cingulate cortex and basal ganglia post-IPT therapy [7]. Goldapple and colleagues discovered metabolic decreases in prefrontal cortical and limbic system activity, but a reversed treatment response pattern in ADM [8]. Kennedy and colleagues then compared the neural correlation of CBT to ADM by fMRI [9] and found similar results. These functional imaging results reveal both common and differential therapeutic pathways between PT and ADM.

White matter abnormalities have been associated with MDD and other psychiatric diseases. Early MRI studies have revealed white matter hyper-intensity in both MDD and geriatric depression [10]–[12]. Diffusion tensor imaging (DTI) is sensitive to microstructural white matter alterations, and DTI has been used for investigating psychiatric disorders over the past few years. In brain tissue, the free diffusion of water molecules is impeded by several biological structures (including macromolecules, filaments, and membranes), producing anisotropy between axial and radial directions. Therefore, fractional anisotropy (FA) has become a useful parameter for assessing the integrity of white matter bundles. Alteration of FA may be due to developmental factors and/or structural features (i.e., axonal disarray, myelin sheath changes) [13]. Earlier studies that focused on late-life depression found decreased FA was primarily found in the frontal lobe [12], [14], temporal region [15], and cingulate cortex [16]. These findings closely correlate to dysfunction in cognitive and emotional networks. Distributed regions of white matter with decreasing FA have been termed “disconnection syndrome” and are hypothesized to play an important role in MDD pathogenesis. However, FA alone does not fully explain the complex white matter changes, especially in cases of increased FA. Combining axial diffusivity (AD) and radial diffusivity (RD) analysis may provide more information about axonal and myelin sheath changes [17].

To date, most DTI findings come from geriatric depression with a handful of studies focused on non-late-life adult MDD. Furthermore, earlier studies used region of interest (ROI) methods, in which the investigator manually drew a prior region for analysis. This subjective method produces a selection bias, and can overlook substantial pathological changes. In contrast, whole brain voxel-based analysis (VBA) is fully automated and therefore can avoid these defects. Moreover, the VBA method is sensitive to FA alterations in the brain. Most importantly, there has been limited research observing how white matter structures change after antidepressant treatment. In order to understand whether white matter microstructures will change in the course of GI therapy, as well as to determine the possible correlations between functional and structural alterations, more evidence is required to corroborate the aforementioned findings. Therefore, in the current study, specific changes in white matter integrity in response to GI psychotherapy are hypothesized to occur in certain cognitive and emotional neural networks.

## Methods

### 2.1 Participants

Forty-five outpatients were recruited at the First Affiliated Hospital at Chongqing Medical University in Chongqing, China. MDD candidates were interviewed by a skilled psychiatrist using the Diagnostic and Statistical Manual of Mental Disorders-IV (DSM-IV) and then enrolled in the MDD group. Depression severity was rated using the 17-item Hamilton Depression Rating Scale (HDRS) on the day of imaging. Participants who met the following criteria were excluded: 1) comorbidity of other psychiatric illnesses or history of symptoms thereof; 2) neurological illness; 3) history of head injury or other traumatic events; and 4) any antidepressant treatment session four weeks prior to initiating treatment in the study. Twenty-two healthy controls (14 females, 8 males) were recruited by advertisement on the medical center campus and nearby communities.

Baseline MRI scans were performed after the initial clinical evaluation. Depression severity was re-evaluated by the 17-item HDRS at the end of therapy and at four weekly follow-up visits. Patients received their second scan at the fifth week. Seven MDDs (n = 7) refused to get MRI scan thus did not participate in the study after the initial clinical evaluation; four MDDs (n = 4) took antidepressants during the experiment; nine MDDs (n = 9) drop out psychotherapy session, another 4 subjects (n = 4) were excluded due to corrupted DTI data by technical failure in either 1st or 2nd scans. Finally 21 MDD subjects (16 females, 5 males) completed the treatment course and two scanning sessions, those 21-subjects-data were fed to analysis. All 21 participants were right-handed. All subjects were given written informed consent, and all protocols of this study were approved by the ethics committee of the First Affiliated Hospital at Chongqing Medical University, Chongqing, China.

### 2.2 Treatment Performances

In the present study, guided imagery (GI) therapy was used to treat the MDD subjects. Mind-body techniques, such as imagery [18], breath work [19], yoga [20], and meditation [21], have demonstrated significant effects in reducing depressive symptoms. The antidepressant effects of GI therapy may result from reduced anxiety and a better sense of control over life stressors [22]. Reduced cortisol levels have been associated with mindfulness-based stress reduction [23]. While regular self-practice of imagery may be helpful in reducing negative emotions [24], GI therapy administered by an experienced psychiatrist is more effective [25]. GI treatment has also shown therapeutic effects in patients with other medical conditions [26].

One experienced psychiatrist administered GI therapy to all 21 MDD subjects. During GI treatment, patients were invited to: (a) perform deep diaphragmatic breathing using the abdomen and diaphragm; (b) perform progressive muscle relaxation exercises involving tension then full relaxation of each muscle group; (c) imagine relaxing natural scenes like landscapes, while paying attention to smells and natural sounds to stimulate the senses; (d) imagine meeting somebody with whom they could share their life situation; and (e) create positive, comforting, and serene images of the hospital context. The therapist intended that patients idealize a space where they could experience ease, safety, refuge, positive images, and freedom from disturbing thoughts. The following statements were used: “…Imagine meeting with somebody with whom you could share your life situation…. What would you like to share…Feel this environment as a place of peace…where you feel protected…where you can renew your energy and your life…. This is calm and safe environment where you feel free and in peace…”.

We had an extra mutual evaluation to the accomplishment of GI psychotherapy after each session. Both patients and psychotherapist rated the therapy process in 5 degree (from ‘not agreed’ to ‘strongly agreed’) by using an in-house questionnaire which includes 4 dimensions: the relaxing quality of the procedure, mental images, musical tone, and musical volume. Between 70% and 88% agreed or strongly agreed about the therapy process.

### 2.3 MRI Acquisition

Images were acquired using a 3.0 Tesla GE scanner (GE Healthcare, Milwaukee, WI) in the First Affiliated Hospital at Chongqing Medical University. High-resolution T1 structure images were acquired first (TR = 8.268 ms, TE = 3.184 ms, flip angle = 13°, FOV = 256×256 mm^2^, voxel size = 0.8594×0.8594×1 mm^3^). Then, diffusion tensor imaging was applied using a spin-echo DTI-Echo Planar Imaging sequence. Thirty contiguous 5-mm slices were acquired in an axial orientation with an in-plane resolution of 0.9375×0.9375 mm^2^ (TR = 8,000 ms; TE = 87.6 ms, flip angle = 90°, FOV = 256×256 mm^2^). A baseline image (b = 0) and 42 different diffusion orientations were acquired with a b-value of 1000.

### 2.4 Data Processing

DTI data were pre-processed under a set of tools provided in The Functional MRI of the Brain (FMRIB) software library (FSL, version 4.1.3; http://fsl.fmrib.ox.ac.uk/fsl/fslwiki). First, head motions and eddy-current distortions were corrected. Second, the non-diffusion images (B = 0) were extracted with a binary mask created. Third, non-brain tissue was striped, and the brains were realigned to the B = 0 images by BET [27]. Fourth, the diffusion tensor model was calculated at each voxel. Fifth, the diagonal elements (λ1, λ2 and λ3), mean diffusivity (MD), and fractional anisotropy (FA) images were calculated [28]. λ1 represents axial diffusivity (AD), and (λ2+λ3)/2 represents radial diffusivity (RD). Sixth, statistical parametric mapping (SPM8; Welcome Department of Cognitive Neurology, London, UK) was used to co-register B0 images to structural T1 images for each subject, and the transforming parameters were applied to the diffusion data. Seventh, each subject’s structural images were normalized to the standard Montreal Neurological Institute (MNI) space by non-linear registration. Eighth, a transformation matrix was applied to co-register images in order to normalize it to the MNI space. Ninth, the resulting images were re-sampled with a voxel size of 2×2×2 mm^3^. Finally, an 8-mm full-width half-maximum Gaussian kernel was used with the FA map to decrease spatial noise and smooth the data.

### 2.5 Statistical Analysis

For comparison between the MDD group and the healthy control group, primarily a whole brain voxel-wise analysis on the FA map was performed using a two-sample *t*-test methods with SPM8. Age and gender were taken into account as nuisance variables. The level of significance was set at a continuous 50 voxels or above (*p*<0.001). To investigate the effects of GI therapy on diffusion tensor parameters, paired *t*-tests were performed between pre- and post-treatment FA maps. The threshold of significance was set at 50 continuous voxels (*p*<0.01). To control of Type I error, multiple comparisons were corrected by AlphaSim correction based on Monte Carlo simulations (http://afni.nimh.nih.gov/pub/dist/doc/manual/AlphaSim.pdf). The correction was carried out in REST software package [29]. Pre-treatment results survived from AlphaSim correction at p<0.05 (combined height threshold p<0.001 and a minimum cluster size = 23, 5000 iterations). Post-treatment results survived AlphaSim correction at p<0.05 (combined height threshold p<0.01 and a minimum cluster size = 43, 5000 iterations). Then clusters showing FA differences at group level were identified as regions of interest (ROI) to extract FA, MD, AD, and RD values. The ROIs constructed from resulting statistic maps, and then extract mean FA, MD, AD, and RD values of the ROIs from each individual’s diffusion parameters’ maps. Those steps were achieved with the SPM extension toolbox MarsBar (http://marsbar.sourceforge.net/).

Statistical analysis for the extracted data (using Student T-tests) and the subjects’ demographic features (for sex ratio using Chi-Square test, the remains using Student T-tests) were performed in SPSS software. In order to label the changes in anatomical locations, the coordinates of the clusters’ peak values are referenced by the MRI Atlas of Human White Matter, 2^nd^ edition [30]. The results are visualized by MRIcron (http://www.mccauslandcenter.sc.edu/mricro/mricron/).

## Results

### 3.1 Demographic and Clinical Features

The MDD (n = 21) and healthy control (n = 22) groups did not differ in age or male/female ratio. All demographic data and clinical features are listed in Table 1. After four weeks of GI therapy treatment in the MDD group, 20 of the 21 MDD subjects exhibited alleviation of depressive symptoms (defined as reduction in HDRS scores above 50%).

**Table 1:** Demographic and clinical features of the MDD and control groups.

	MDD	Control	*P*-value
**Gender (female:male)**	16∶5	14∶8	0.381#
**Age**	29.6±12.6	30.2±10.2	0.875+
**Illness duration**	14.2±13.4	N/A	N/A
**HDRS score (pre-PT)**	20.4±6.3	2.2±1.6	N/A
**HDRS score (post-PT)**	4.4±3.7	1.0±1.7	N/A

# chi-square test;

+ two-sample t-test;

HDRS: 17-item Hamilton Depression Rating Scale; PT: psychotherapy.

### 3.2 Analysis of Diffusion Data

**Figure 1:**
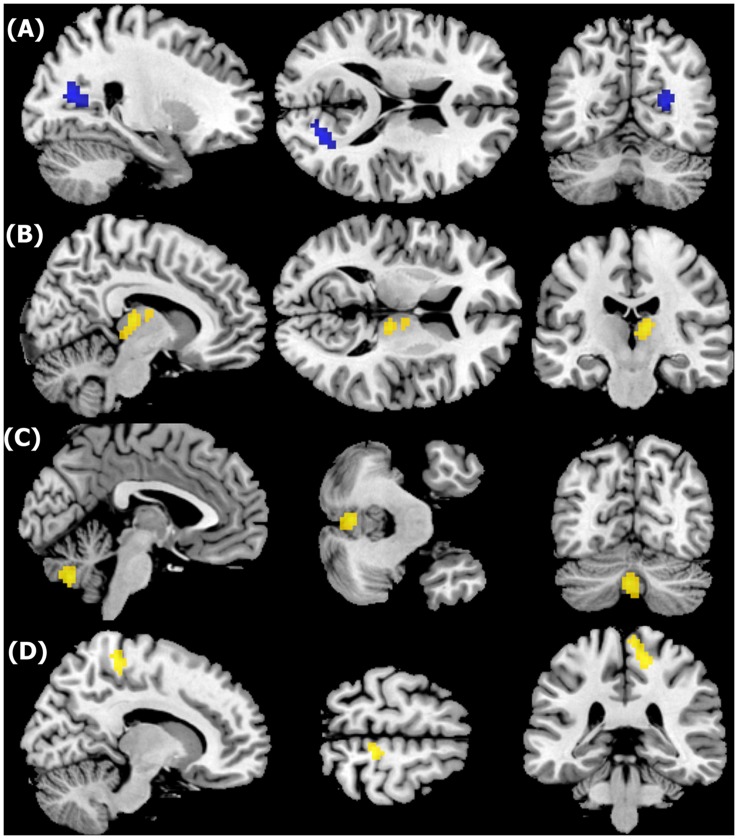
Fractional anisotropic (FA) changes in MDD subjects relative to healthy controls, shown through sagittal, transverse, and coronal views (*p*<0.001, uncorrected). (A): Decreased FA in the right cuneus gyrus white matter. (B)(C)(D): Increased FA in the right thalamus, right postcentral gyrus, and cerebellar vermis, respectively.

Compared to the healthy control group, the MDD group exhibited aberrant FA values in four brain regions (p = 0.05 corrected, Fig. 1 and Table 2). Significant decreases in FA for MDD subjects were observed in the right cuneus gyrus, together with decreases in AD (*p* = 0.013) and MD (*p* = 0.037) and increases in RD (*p* = 0.000). Increases in FA were observed within three clusters: i) the right thalamus displayed increase in AD (*p* = 0.031) but decrease in MD (*p* = 0.002) and RD (*p* = 0.000); ii) the right postcentral gyrus displayed decrease in AD (*p* = 0.002), MD (*p* = 0.001), and RD (*p* = 0.001); and iii) although a significant increase in FA was seen in the cerebellar vermis, no significant alterations in AD (*p* = 0.398), MD (*p* = 0.152), and RD (*p* = 0.080) were found.

**Table 2:** Fractional anisotropy alterations in the MDD and healthy control groups.

Location	Peak MNI Coordinate	# of Voxels	*P*-value (Corrected)
**Pre-treatment (MDD group versus healthy control group)**			
Right thalamus	9, −21, 9	76	0.05
Right postcentral gyrus	12, −36, 63	74	0.05
Cerebellar vermis	−3, −69, −36	53	0.05
Right cuneus gyrus	24, −66, 15	75	0.05
**Post-treatment changes**			
Left supplementary area	−6, 6, 52	57	0.05
Right angular gyrus	40, −62, 36	78	0.05

**Figure 2:**
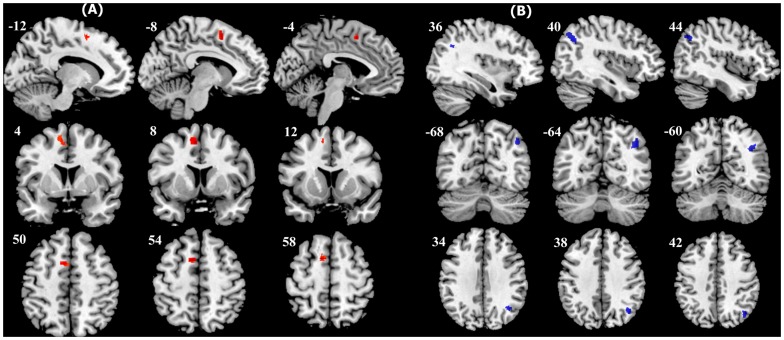
Fractional anisotropic (FA) changes after four weeks of GI therapy, shown through sagittal, transverse, and coronal views using a four-voxel interval (*p*<0.01, uncorrected). (A): Increased FA in the left supplementary area. (B): Decreased FA in right angular gyrus.

Treatment effect shows aberrant FA values in two regions (Fig. 2 and Table 2): i) increased FA in the left superior frontal cortex (supplementary motor area/SMA) and ii) decreased FA in the right angular cortex white matter (p = 0.05 corrected). In the frontal cluster, AD (*p* = 0.815), MD (*p* = 0.499), and RD (*p* = 0.354) did not differ from each other; in the other cluster, increased MD and RD were found (two-tailed paired *t*-test, *p* = 0.001). In addition to ROI analysis, Pearson correlation between mean FA values in significant clusters and depression severity (HDRS scores)/HDRS reduction-rate were calculated. Post-treatment FA in frontal cluster moderately correlated to HDRS (r = 0.494, p = 0.023) and reduction-rate (r = −0.504, p = 0.020), but not in the cluster of right angular cortex (Table 3).

**Table 3:** Correlations between pre-post FA and HDRS and HDRS reduction-rate.

	HDRS	p- values	Reduction-rate	p-values
Left SMA Pre-PT	0.084	0.717	−0.328	0.147
Post-PT	0.494	0.023*	−0.504	0.020*
Right AG Pre-PT	0.386	0.084	−0.128	0.579
Post-PT	0.031	0.893	0.104	0.084

* Correlation is significant at the 0.05 level (2-tailed); AG = Angular Gyrus.

## Discussion

This is the first study to assess white matter microstructural changes pre- and post-PT. First, decreased FA was not only found in the white matter, but increased FA was also observed across several widespread brain regions. Second, four weeks of PT produced changes in white matter’s diffusion characteristic. Post-treatment changes did not take place in existing abnormal regions, which suggests that GI therapy does not directly correct the underlying psycho-pathophysiology, but rather remodels mood regulation networks and boosts cortical control.

It is generally believed that the microstructural disruption in white matter, damaged axonal membranes, and demyelination gives rise to decreased FA and increased regional water diffusion [17]. However, the nature of the low FA value found in white matter has not been clearly elucidated at the subcellular level. Nevertheless, several studies have revealed significant increases in FA value [31], [32], suggesting that the impacted white matter bundles represent a pathological condition. In spite of the varying methods used in DTI analysis (ROI, VBA or TBSS), decreased FA has been observed mainly in unipolar depression, yet changes in two different directions coexist in bipolar disorder [33]. Both mood disorders share some common symptomatic features, as they represent the two extreme mood states in the affective disorder spectrum [34]. Another possible interpretation is that the higher FA appears in a specific disease stage, in which both lower and higher FA are expressed concurrently in different brain regions.

The thalamus is a structure which develops from the embryonic diencephalon, and its anterior portion belongs to the limbic system. Postmortem investigation has discovered elevated neuron counts in the thalamus [35]. Previous studies have revealed altered FA in the thalamus [36]. Greicius et al. also found increased thalamic functional connectivity in the default mode network of depressed subjects [37]. The thalamus serves as a hub that interconnects cortical and subcortical nuclei by a significant number of fibers. The medial dorsal thalamus nucleus reciprocally connects to the dorsolateral prefrontal cortex (DLPFC), the orbitofrontal cortex (OFC), and the anterior cingulate cortex (ACC) [38]. Disruptions between the cortex and thalamus were found by fMRI and DTI in MDD [39]–[41]. In addition, the medial prefrontal cortex and adjacent regions together are collectively known as the medial prefrontal cortex (mPFC) network. The mPFC network is connected to the medial thalamus, and this system is involved in modulating visceral function in animal models and human mood disorders [42]. Convergent data indicates disrupted neuronal circuits in the limbic–thalamic–cortical (LTC) and limbic-cortical-striatal-pallidal-thalamic (LCSPT) regions in MDD [43]. These networks are related to emotional behavior and visceral function due to their substantial links with the hypothalamus and periaqueductal gray matter (PAG) [6]. The hypothalamic-pituitary-adrenal (HPA) axis is hyperactivated by stress [44], and its dysfunction has been correlated with depression. The hyperactivated HPA axis upregulates the release of corticotropin-releasing hormone and cortisol, which has been detected in the cerebrospinal fluid and plasma of MDD patients [45], [46]. With wide-ranging connections across several cortical-subcortical regions, the thalamus serves as a “relay station” in the affective network. In the context of MDD, elevated thalamic FA indicates disrupted cortical regulation and poorly controlled limbic functioning, thereby generating and sustaining a depressive mood state.

The present findings reveal that GI therapy increases FA within the left superior frontal gyrus – specifically, the supplementary motor area (SMA), Brodmann Area 6. The vertical commissure anterior line divides the SMA into two parts: i) the pre-SMA rostrally and ii) the remaining SMA caudally. Functional connectivity studies have also indicated that the pre-SMA area is involved in cognitive functioning [47]. The SMA directly connects to the corticospinal tract and motor neurons; however, the connectivity pattern in the pre-SMA is quite different. The pre-SMA has reciprocal connections with the DLPFC [48] and ACC [49], which are associated with cognitive control/executive functioning and conflict monitoring [50], respectively. BA 6 influences cognitive control in the medial-lateral frontal cortex [51]. Moreover, pre-SMA has been shown to contribute more to conflict monitoring than the ACC [52]. The OFC is crucial to emotional regulation circuitry. Two studies have demonstrated functional connectivity between the OFC [53] and the left SMA [54] in MDD patients. Elevated white matter integrity may not only reverse these deficits, but may also enhance coupling between the pre-SMA, cognitive control in the frontal lobe, and affective networks in the frontal-striatal circuitry. Attenuated cognitive control commonly accompanies depression through increasing negative appraisals and sustaining pessimistic mood states [55]. From the cognitive model perspective of depression, individuals with biased schema are prone to deviate from normal information processing, thereby increasing their vulnerability to internal and external negative stimuli [4]. Through positive scene visualization, GI therapy may enable the reconstruction of connections between information processing centers and their regulative networks, thereby correcting depressive schema by helping patients to better “filter” negative thoughts.

The improved white matter integrity in SMA is correlated with depressive symptom remission, but not with severity. The SMA is probably not directly related to the onset of depression, since it is not in the primary subcortical-limbic emotion-mood circuitry. As discussed above, the observed post-treatment changes might result from remodeling of frontal mood-regulatory network. Although the limbic emotional circuitry is modulated by frontal networks, it is noteworthy that after four weeks of GI therapy, the existing pre-treatment white matter disruptions have not been normalized. Rather, Goldapple et al.’s work suggests frontal activity is reserved for recruitment of cognitive control post-treatment [8]. The present findings relate to the proposed “top-down” [3] mechanism of PT, as previous observations have been localized in frontal mood-related networks. Treatment effects lead to new and enhanced connections between certain functional nodes, which alters local properties and remodels global emotion-related architecture. Modulated cortical information processing and facilitated cognitive control over the limbic system may attenuate depressive mood states. Changes in the dynamic interactions between “upper” cognitive networks and “lower” emotional networks are likely responsible for the efficacy of PT. In support of this point, metabolic changes in sub-cortical and limbic regions were found in a previous 16-week CBT trial [9]; however, these changes were not found here. This discrepancy may be due to the differing imaging and therapeutic modalities employed, especially the assessment at a rather late time-point. The microstructural white matter changes were purposely examined in patients of four-week treatments, as the neural response to GI therapy was likely initiated in frontal networks. This outcome suggests a “top-down” pattern analogous to the functional results. Comparing PT to ADM, the two remedies show differing patterns in treatment response, but overlapping alterations in common pathogenic pathways and normalizing processes in neural circuitry. Interestingly, both electroconvulsive therapy [15] and transcranial magnetic stimulation [56] have demonstrated increased FA in the frontal lobe, suggesting that frontal network remodeling is not unique to GI therapy.

There are several limitations to this study. First, the sample size was relatively small, and subjects’ ages and illness durations were not completely homogeneous. Second, the identical control group was not rescanned after a similar 4 weeks interval, thereby confound variables might be involved with either time effect or imaging reproducibility. Third, the fact that the enrolled MDD subjects voluntarily chose GI therapy may indicate a psychotherapy-prone bias, which may partially contribute to the high recovery rate observed in this study. Fourth, further neuropsychological testing was not performed to further support the speculated behavioral and cognitive defects.

In conclusion, alterations in the white matter microstructure have been demonstrated within the sub-cortical, cortical areas, and cerebellum of MDD subjects relative to healthy controls. Post-treatment effects exhibit an increase in FA within the left frontal lobe, specifically a portion of the SMA and adjacent regions. These findings support the proposed “top-down” mechanism of PT.
